# Supplementary material for: Sorting at embryonic boundaries requires high heterotypic interfacial tension
Source: Nat Commun. 2017 Jul 31;8:157. doi: 10.1038/s41467-017-00146-x (PMC5537356; doi:10.1038/s41467-017-00146-x)
Supplement: Supplementary file 2 — Supplementary Software 1 [file 41467_2017_146_MOESM2_ESM.zip › PottsModel/SrcPottsModel/doc/overview-tree.html]

Class Hierarchy


JavaScript is disabled on your browser.


Skip navigation links


- Overview
- Package
- Class
- Use
- Tree
- Deprecated
- Index
- Help

- Prev
- Next

- Frames
- No Frames

- All Classes

# Hierarchy For All Packages

Package Hierarchies:

- engine,
- gui,
- model,
- mvc,
- simulations,
- utils

## Class Hierarchy

- java.lang.Object
  - mvc.AObservable (implements mvc.IObservable)
    - model.Cell (implements model.KnowsConstants)
      - model.SquareCell
    - model.Lattice (implements java.lang.Iterable<T>, model.KnowsConstants, mvc.Observer)
    - engine.PottsEngine (implements model.KnowsConstants)
    - engine.StatisticsManager (implements mvc.Observer)
  - model.AreaEnergyFunction (implements model.EnergyFunction)
  - model.CartesianCoordinates (implements model.Coordinates)
  - gui.CellDisplay
  - model.CellSet (implements java.lang.Iterable<T>)
  - utils.ColorScale
  - engine.CommandLineSimulation
  - java.awt.Component (implements java.awt.image.ImageObserver, java.awt.MenuContainer, java.io.Serializable)
    - java.awt.Container
      - javax.swing.JComponent (implements java.io.Serializable)
        - javax.swing.JPanel (implements javax.accessibility.Accessible)
          - gui.ConfigurationInformationPanel (implements gui.EngineObserverPanel)
          - gui.ConfigurationPanel
          - gui.Console
          - gui.PlotPanel (implements mvc.Observer)
          - gui.PottsCanvas (implements mvc.Observer, java.beans.PropertyChangeListener)
          - gui.PottsToolbar
          - gui.StatusBar (implements gui.EngineObserverPanel, mvc.Observer)
      - java.awt.Window (implements javax.accessibility.Accessible)
        - java.awt.Frame (implements java.awt.MenuContainer)
          - javax.swing.JFrame (implements javax.accessibility.Accessible, javax.swing.RootPaneContainer, javax.swing.WindowConstants)
            - gui.PottsFrame (implements java.awt.event.ActionListener, mvc.Observer)
  - gui.ConfigurationStartMenu
  - model.Constants
  - model.Constants.ParameterManager
  - model.EnergyManager
  - model.FiniteVector (implements model.Vector)
  - model.HalfLine (implements model.Vector)
  - gui.Hexagon (implements gui.PixelShape)
  - gui.Hexagon.AxialCoordinates (implements model.Coordinates)
  - gui.Hexagon.CubeCoordinates (implements model.Coordinates)
  - gui.Hexagon.OffsetCoordinates (implements model.Coordinates)
  - model.HexagonPositionManager (implements model.IPositionManager)
  - utils.HSLColor
  - model.InterfacialTensionEnergyFunction (implements model.EnergyFunction)
  - utils.IO
  - utils.JavaVersion
  - model.Lattice.PixelIterator (implements java.util.Iterator<E>)
  - model.LatticeUtilities
  - model.Pair<A,B>
  - model.PerimeterEnergyFunction (implements model.EnergyFunction)
  - model.Pixel (implements model.KnowsConstants)
  - gui.PixelDisplay
    - gui.HexagonPixelDisplay
    - gui.SquarePixelDisplay
  - model.PixelInteractionEnergyFunction (implements model.EnergyFunction)
  - engine.PottsEngine.StateVariables
  - engine.PottsLogger (implements mvc.Observer)
  - engine.Simulation
    - simulations.BoundarySimulation
    - simulations.DefaultSimulation
    - simulations.DITHSimulation
    - simulations.EndoSimulation
    - simulations.FourByFourSimulation
    - simulations.GenerateRandomLattices
    - simulations.GranerGlazierSimulation
    - simulations.NegativeControl
    - simulations.SingleCell
  - gui.SnapshotManager (implements mvc.Observer)
  - gui.Square (implements gui.PixelShape)
  - model.SquarePositionManager (implements model.IPositionManager)
  - engine.Statistic
    - engine.CellEnergyStatistics
    - engine.CellStatistic
      - engine.AreaEnergyStatistic
      - engine.AreaStatistic
      - engine.InteractionEnergyStatistic
      - engine.PerimeterStatistic
    - engine.CSVStatistic<LabelEnum>
      - engine.CellCoordinatesCSVStatistic
      - engine.CellShapeCSVStatistic
    - engine.DispersionIndex
    - engine.EnergyStatistic
    - engine.HBLStatistic
    - engine.TypeSpecificCellStatistic
      - engine.HMDStatistic
      - engine.IsoperimetricQuotientStatistic
      - engine.TypeSpecificAreaStatistic
      - engine.TypeSpecificNearestNeighborStatistic
      - engine.TypeSpecificNumNeighborsStatistic
      - engine.TypeSpecificPerimeterStatistic
    - engine.TypeSpecificStatistic
      - engine.TypeSpecificPercentIsolatedCellStatistic
  - engine.Statistic.Utils
  - engine.Utils
  - gui.Utils
  - engine.Utils.EnergyTracker
  - model.VectorTests
  - model.VectorUtils

## Interface Hierarchy

- model.Coordinates
- model.EnergyFunction
- gui.EngineObserverPanel
- model.IEnergyFunction
- mvc.IObservable
- model.IPositionManager
- model.KnowsConstants
- mvc.Observer
- gui.PixelShape
- model.Vector

## Enum Hierarchy

- java.lang.Object
  - java.lang.Enum<E> (implements java.lang.Comparable<T>, java.io.Serializable)
    - engine.PottsEngine.State
    - engine.CellShapeCSVLabel
    - model.Pixel.PixelType
    - model.Lattice.Type
    - model.Lattice.State
    - model.IPositionManager.Position
    - model.EnergyFunction.Energy
    - model.Constants.Model
    - model.Cell.CellType
    - model.Cell.Energy
    - model.Cell.State
    - gui.PottsFrame.Action
    - gui.PixelShape.Edge
    - gui.PixelShape.Type

Skip navigation links


- Overview
- Package
- Class
- Use
- Tree
- Deprecated
- Index
- Help

- Prev
- Next

- Frames
- No Frames

- All Classes
